# Successful use of conjunctival flaps to prolong survival of type I keratoprosthesis in severe bilateral chemical burns: two case reports

**DOI:** 10.12688/f1000research.131492.1

**Published:** 2023-05-19

**Authors:** Sheetal Mahuvakar, Neha Jain, Sayan Basu

**Affiliations:** 1 Cornea and Refractive Services, Dr. Nagpal's Retina Foundation, Ahmedabad, Gujarat, India; 2 Shantilal Shanghvi Cornea Institute, LV Prasad Eye Institute, Hyderabad, Telangana, 500034, India; 3 Prof. Brien Holden Eye Research Centre (BHERC), LV Prasad Eye Institute, Hyderabad, Telangana, 500034, India

**Keywords:** Chemical injury, Bilateral limbal stem cell deficiency, Keratoprosthesis, Type I keratoprosthesis

## Abstract

Introduction:

This report describes the use of conjunctival flaps to enable the survival of type I keratoprosthesis (KPro) in two cases of bilateral severe total limbal stem cell deficiency (LSCD) following chemical burns.

Presentation of case:

Two patients had a history of bilateral chemical injury with lime. On examination, the presenting vision was light perception to hand motions and both cases had conjunctivalized ocular surfaces with symblepharon. A modified technique of type I keratoprosthesis was used, where the conjunctivalized corneal pannus was dissected and lifted off as an inferior fornix-based conjunctival flap. This was followed by a standard surgical technique of type I KPro. The flap was then secured over the device and optical opening was made two weeks later. Both the patients had stable ocular surfaces with visual acuity of 20/20 at 2–7 years of follow-up.

Discussion:

In patients with total LSCD with adnexal involvement, type I KPro has unsatisfactory long-term survival because of the risk of repeated epithelial breakdowns and stromal ulceration. With the innovative approach described in this report, type I KPro can be successfully used for sustainable visual improvement in the presence of severe ocular surface disease and symblepharon.

Conclusion:

Conjunctival flaps can be used along with type I KPros to improve long-term survival and give sustainable visual outcomes in cases of bilateral corneal blindness due to advanced ocular surface damage.

## Introduction

A keratoprosthesis (KPro), or artificial corneal prosthesis, is preferred for visual rehabilitation in total limbal stem cell deficiency (LSCD) and corneal opacification, where penetrating corneal grafts are unlikely to survive long term due to the presence of extensive stromal vascularization, and poor ocular surface. ^1^^–^^3^ The type I KPro is used in wet eyes with normal lid anatomy and blink function. ^4^ With continuous modification in the structure of the type I device, complications like retro-prosthetic membrane, extrusion, *etc.* have reduced especially for non-immune etiology. ^2^^,^^5^ To prevent recurrent epithelial breakdown over the donor corneal graft and sterile corneal necrosis, a soft bandage contact lens (BCL) is placed after the KPro surgery. ^6^^,^^7^ In patients with dry eyes, abnormal lid function, extensive keratinization or symblepharon, such as in Stevens-Johnson syndrome (SJS), mucous membrane pemphigoid (MMP) or severe ocular burns where the risk of corneal graft melt is high or it is difficult to retain a BCL, a more complicated type II KPro needs to be performed. ^4^^,^^5^ Thus, to overcome the limitations of a type I KPro in severe ocular surface disease and avoid the need for a type II KPro, we describe a new approach of type I KPro in two cases of chemical injury sequelae with bilateral total LSCD with corneal pannus and extensive ocular surface involvement. This report is as per the SCARE-2020 guidelines. ^8^

## Case series

### Case 1

A 26-year-old male student from the Indian north-east presented with a history of chemical injury with lime in both eyes one year ago. There was no relevant family history. An amniotic membrane grafting (AMG) with symblepharon release was done in both eyes at another hospital. At presentation, visual acuity was hand motions in both eyes. On examination, lid margin in both eyes were thickened with diffuse bulbar conjunctival congestion and 360 degree corneal conjunctivalization. In the right eye, symblephara were present superiorly and temporally. In the left eye, symblepharon were present temporally. A pyogenic granuloma was present at 1 o’clock with a descemetocele in the visual axis in the left eye. The rest of the anterior segment details could not be visualized. Both eyes had some wetness, but the Schirmer’s test was recorded as 5 mm of wetting at 5 minutes. Digital intra-ocular pressure (IOP) and B-scan was normal in both eyes. He was diagnosed with bilateral total LSCD with left eye descemetocele. A type I KPro implantation was planned for the right eye. Because of the severity of the ocular surface damage and the high risk of post-operative surface complications a different approach was taken.

The surgery was done under local anesthesia. Conjunctival peritomy was done superiorly. The conjunctivalized pannus covering the corneal surface was separated from the underlying adhesions by blunt dissection and lifted off the cornea as a single flap with an inferior hinge. Excess fibrovascular proliferation in the undersurface of the reflected flap was excised to make it thin. The host cornea was then trephined with 8.5 mm hand-held trephine and donor cornea with 9.0 mm hand-held trephine. Aurolab keratoprosthesis of +59.0 dioptre was assembled. the graft–host junction was sutured with 16 interrupted sutures with 9-0 nylon **.** The pannus was replaced back and secured into position with fibrin glue (Tisseel Kit, Baxter AG, Vienna, Austria).

**Figure 1.**
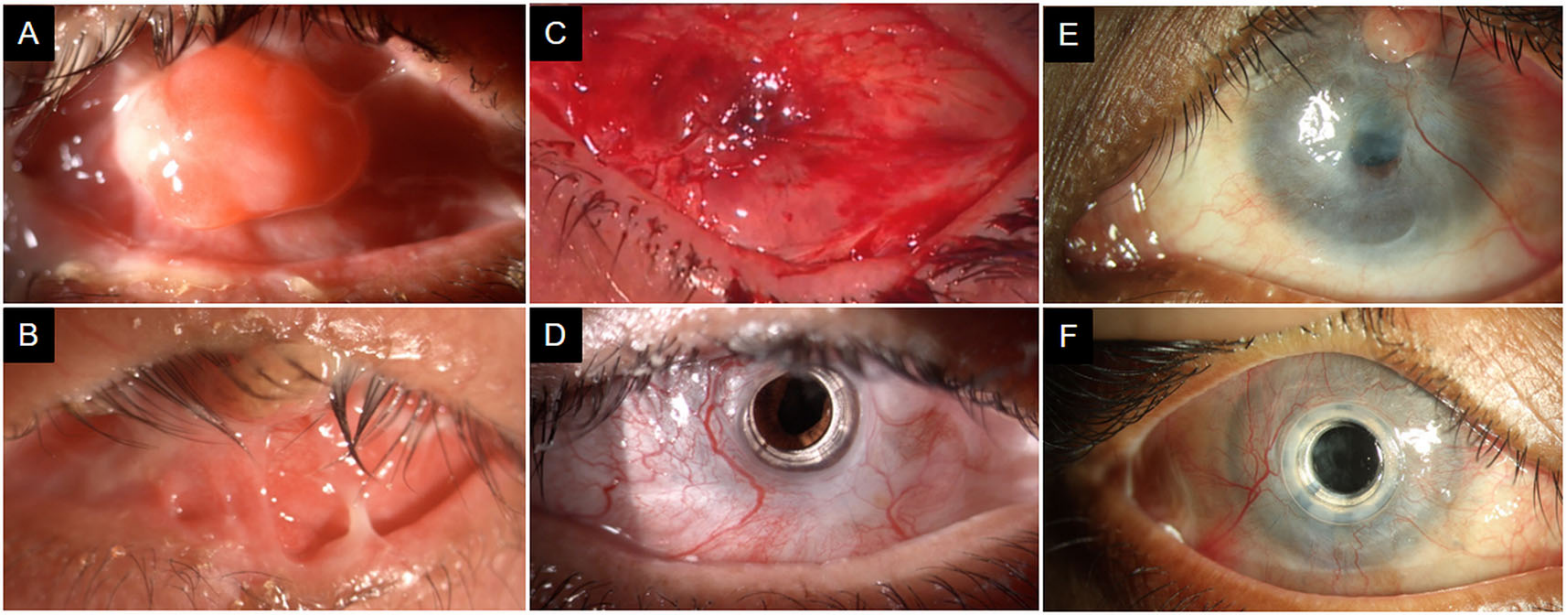
This is a collage of images of both the patients before and after the surgery. (A) The right eye of the first patient with total limbal stem cell deficiency with granuloma over the cornea. Superior and inferior symblephara are present. Anterior segment cannot be visualized. (B) Left eye of the same patient with distichiasis and trichiasis in upper and lower lid. Granuloma is present in the central upper lid. Total limbal stem cell deficiency in present with extensive symblephara. (C) In the right eye, the area of accidental cut supero-nasally was meticulously sutured. The area has healed well with no defect. (D) Right eye of the first patient, 3 days after the central opening was made. The carrier graft is conjunctivalized. The keratoprosthesis is in place with clear visual axis. The peripheral iridotomy can be seen supero-nasally. (E) The left eye of the second patient. Symblephara are present temporally. Cornea in conjunctivalized completely with a descemetocele in the visual axis. Pyogenic granuloma is present at 1 o clock. The right eye of the patient had similar clinical features on presentation. (F) Right eye of the second patient at 15 months post-surgery. The keratoprosthesis is in place with the carrier graft well conjunctivalized. The ocular surface with healthy with no corneal melt. The visual axis is clear with mild posterior capsular opacification.

**Figure 2.**
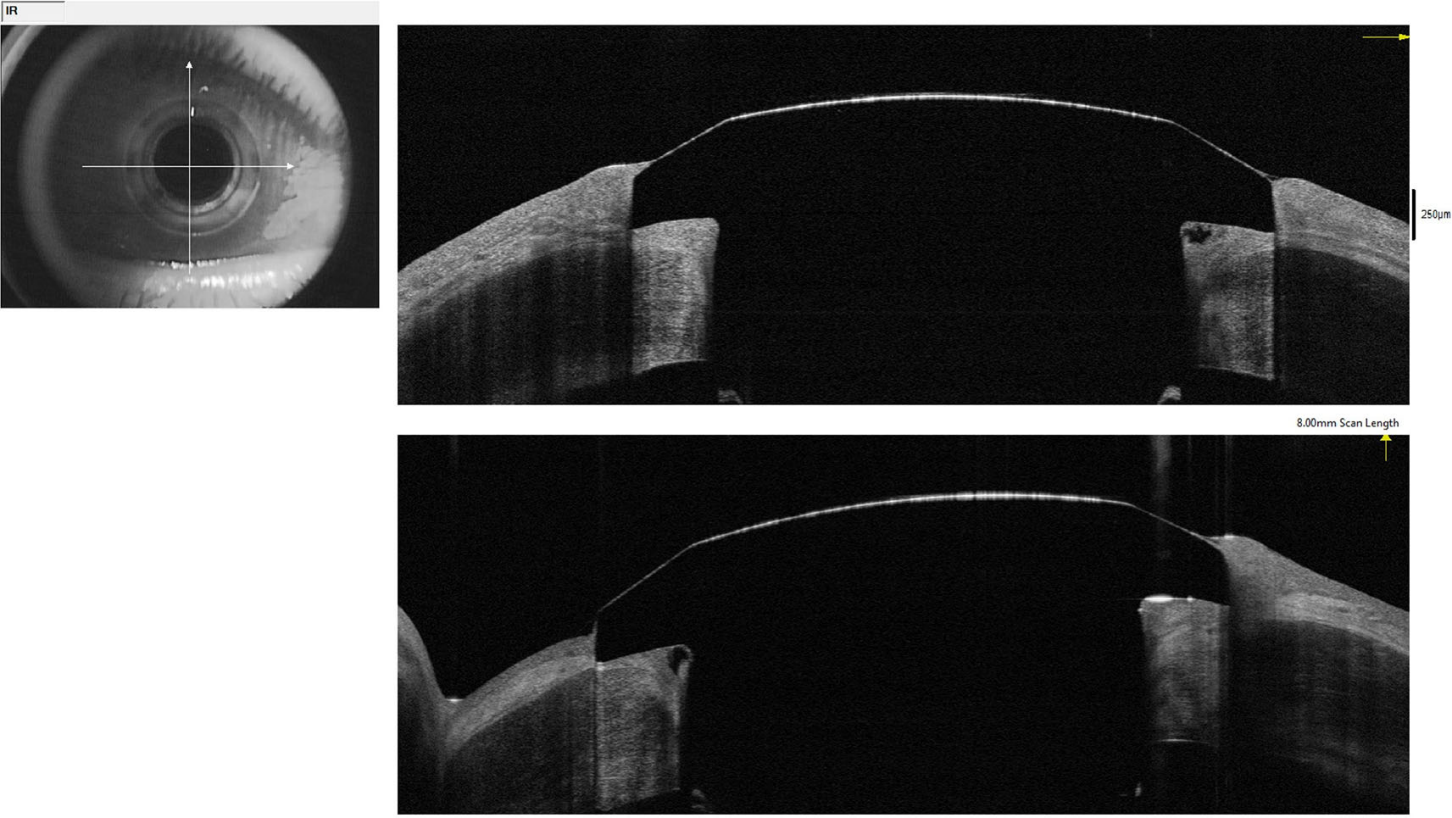
Anterior segment optical coherence tomography image at 82 months of follow-up showing a conjunctivalized surface with a stable type 1 keratoprosthesis in place.

On first post-operative day, mild lid edema was present along with diffuse conjunctival congestion. The anterior surface of the keratoprosthesis was covered by pannus. The patient was started on topical corticosteroid (prednisolone acetate 1%, 6 times per day), antibiotic (moxifloxacin 0.5%, 4 times per day), anti-glaucoma (timolol 0.5%, twice daily) eyedrops along with oral acetazolamide 250mg (once daily at bedtime). An opening in the conjunctival flap, just large enough to expose the underlying optical cylinder, was made 2 weeks later after which the vision improved to 20/50P. The keratoprosthesis was present in place and the ocular surface was stable. Fundus examination was normal. At last follow-up at 82 months, visual acuity in the right eye with −1.25D of spherical correction was 20/20. The ocular surface was stable with conjunctivalized corneal graft and keratoprosthesis in place ( Figure 1, 2). Mild posterior capsular opacification was noted. Topical corticosteroids were continued along with topical antibiotic and anti-glaucoma eyedrop.

### Case 2

A 45-year-old male carpenter from north India presented with a history of injury to both eyes with toilet cleaning solution two months ago. There was no relevant family history. He had undergone AMG twice at another hospital. At presentation he had total LSCD with persistent epithelial defects (PED) in both eyes for which AMG with tarsorrhaphy was repeated. Over 3 months, thick fibrotic pannus with granuloma covering cornea and symblepharon was noted in both eyes. Living-related allogeneic simple limbal epithelial transplantation (SLET) was done in the right eye which eventually failed. A Boston type I KPro was done for the left eye which later developed microbial keratitis due to which it had to be explanted.

After 9 months, visual acuity was light perception with accurate projection of rays in both eyes. On slit lamp examination, both eyes had thickened lid margins and distichiasis. Both eyes had thick fibrotic pannus over the cornea, with superior and inferior symblepharon. A granuloma covering the cornea was present in the right eye ( Figure 1). Both eyes were relatively wet. B-scan in both eyes had anechoic vitreous. He was planned for a type I aphakic KPro implantation in the right eye. However, with the hindsight of epithelial healing issues and infection in the left eye, and the encouraging outcome seen in the previous case, a decision to use a conjunctival flap was made.

The surgery was done under local anesthesia. The conjunctival flap was raised in a manner similar to the previous case, however there was an inadvertent buttonhole created supero-nasally. Host cornea was trephined with 8.75 mm hand-held trephine and removed. Open sky cataract extraction was performed leaving intact posterior capsule. Two peripheral button iridectomies were done in superior quadrant. Donor cornea was trephined with 9 mm hand-held trephine followed by central 3 mm trephination and Aurokpro was assembled. The graft–host junction was sutured with 16 interrupted sutures with 10-0 nylon. The pannus was replaced in its original position covering the whole ocular surface including KPro and sutured to episcleral tissue with 8-0 vicryl sutures. The inadvertent cut was also sutured meticulously.

On the first post-operative day, topical antibiotic (gatifloxacin 0.5%, 4 times per day) was started along with anti-glaucoma (timolol maleate + brimonidine tartarate, twice per day) eyedrops. Topical corticosteroids (prednisolone acetate 1%, 6 times per day) were started and tapered over 3 weeks to a maintenance dose of 3 times per day. An optical opening was made after 12 days following which the vision was 20/40. After 4 months vision dropped to 20/120. On examination, posterior capsular opacification was noted. On OCT, macular edema was seen. YAG capsulotomy was done along with sub-tenon’s triamcinolone acetanoid injection. At the last follow-up at 24 months, vision was 20/20. On examination, the KPro was noted to be in place and the ocular surface was stable. Resolution of macular edema was seen on OCT. Topical corticosteroid, antibiotic and anti-glaucoma eyedrop were continued.

## Discussion

In cases with severe ocular surface disease and adnexal involvement, the risk of surface breakdown, peri-optical cylinder melting, and infection is high with the use of the type I KPro. It is also often difficult to retain the BCL because of symblephara and forniceal shortening, which further contributes to the threat of surface complications. These cases are more amenable to the type II KPro. ^4^^,^^5^ However, the surgical procedure of the type II KPro is complex and carries many disadvantages like poor cosmesis, restricted visual fields, in addition to complications of type I KPro. A multidisciplinary team is required as vitrectomy and glaucoma surgery is done concurrently, restricting it’s availability at few centers worldwide. ^9^^,^^10^ To overcome these disadvantages, the surgical technique of type I keratoprosthesis was modified to improve the survival in eyes with cicatrizing ocular surface disease that are prone to sterile corneal melting.

The use of conjunctival flaps in KPros in not new. Conjunctival flap was reported to be mobilized and positioned on the KPro followed with delayed opening. ^11^^–^^13^ In another technique for patients with inferior fornix foreshortening, the superior tarsal and palpebral conjunctiva was mobilized to cover the KPro entirely and optical opening was made 3 months later. Although the KPro was retained on long-term follow-up, it predisposed the patients to develop ptosis due to advancement of forniceal tissue over the cornea. ^5^ Conjunctival flap or oral mucous membrane graft has also been used to salvage an isolated area of device exposure. ^14^^,^^15^ In all above techniques, the conjunctiva was freely mobile and could easily cover the device entirely.

In the present cases, along with total LSCD, ocular surface was extensively involved with conjunctival cicatrization, granuloma, and symblepharon. The above-mentioned techniques of conjunctival mobilization were not possible due to extensive adhesions as it would have been difficult to advance the conjunctiva to cover the device entirely. This report describes a novel way of using conjunctival coverage to overcome the possibility of sterile keratolysis and eliminate the need for BCL usage, while retaining the technical ease of implanting a type I KPro. The advantage is that it can be done in cases where symblepharon and fibrosis restricts the advancement of conjunctiva. Repositioning of the conjunctivalized corneal pannus over the KPro helps in faster conjunctivalization of the carrier graft, thus increasing the retention of the KPro and decreasing the probability of extrusion and corneal melt despite avoiding the use of a BCL. As lid anatomy is not distorted, there is no possibility of ptosis later. The surgery is comparatively simpler and less disfiguring, thus can have more widespread acceptability as well.

## Conclusion

In chemical injury sequelae with ocular surface involvement, type I KPro implantation under the corneal pannus followed by creation of an optical opening, provides a stable ocular surface and sustainable visual recovery in the long-term.

## Consent

Written informed consent was obtained from the patients for publication of this case series and accompanying images.

## Ethical approval

Ethics committee approval was not required for this manuscript because it is a clinical case report

## Author contribution

Study concept or design: SM, SYB

Writing and revising the paper: SM, SYB, NJ

## Research registration

Not applicable

## Data Availability

All data underlying the results are available as part of the article and no additional source data are required.
