# Supplementary material for: Successful use of conjunctival flaps to prolong survival of type I keratoprosthesis in severe bilateral chemical burns: two case reports
Source: F1000Res. 2023 May 19;12:521. [Version 1] doi: 10.12688/f1000research.131492.1 (PMC10618640; doi:10.12688/f1000research.131492.1)
Supplement: Peer review history

# Reviewer report: Reviewer response for version 1

**DOI:** 10.5256/f1000research.144340.r214602

**Published:** 2023-10-31

**Authors:** Jiaqi Chen

**Affiliations:** 1 Zhongshan Ophthalmic Center, Sun Yat-sen University, Guangzhou, China

This article reports the use of conjunctival flaps in patients with type I KPro implantation. This surgical procedure is useful and enables  patients with symblepharon and forniceal shortening to receive a type I KPro, which is usually indicated for the type II KPro.

 In patient 2, the symblephron is comparatively modest from the images. One alternative method may be the symblepharon lysis and buccal mucosa implantation to reconstruct the ocular surface as we used before, which may ameliorate the blink-related microtrauma to the cornea and chronic inflammation (Zhang *et al.*, 2020 ^1^).

Are enough details provided of any physical examination and diagnostic tests, treatment given and outcomes?

Yes

Is the background of the cases’ history and progression described in sufficient detail?

Yes

Is sufficient discussion included of the importance of the findings and their relevance to future understanding of disease processes, diagnosis or treatment?

Partly

Is the conclusion balanced and justified on the basis of the findings?

Yes

Reviewer Expertise:

Cornea disease

I confirm that I have read this submission and believe that I have an appropriate level of expertise to confirm that it is of an acceptable scientific standard.

## References

[1] : Treatment of Oculoplastic and Ocular Surface Disease in Eyes Implanted with a Type I Boston Keratoprosthesis in Southern China: A Retrospective Study. *Adv Ther* .2020;37(7) : 10.1007/s12325-020-01381-3 3206-3222 10.1007/s12325-020-01381-3 32436025

# Reviewer report: Reviewer response for version 1

**DOI:** 10.5256/f1000research.144340.r183670

**Published:** 2023-07-27

**Authors:** Amit Raj, Prabhakar Singh

**Affiliations:** 1 All India Institute of Medical Sciences, Patna, Bihar, India; 2 Cornea and anterior segment services, Ophthalmology, All India Institute Medical Sciences, Patna, Patna, Bihar, India

Congratulations to the authors for giving insight into the use of conjunctival flap in cases of type 1 Keratoprosthesis. Definitely, the procedure gives an added advantage of a more stable ocular surface with lesser surface-related complications; however, the absence of a flange/collar in the anterior-most part of the optic results in repeated conjunctival growth over the optic obscuring visual axis. This needs frequent excision of the overgrown conjunctiva. This complication can be overcome with designs of type 1 keratoprosthesis with an added flange/collar. The conjunctiva can be tucked in beneath this collar/flange with other advantages and ease of type 1 keratoprosthesis.

Are enough details provided of any physical examination and diagnostic tests, treatment given and outcomes?

Yes

Is the background of the cases’ history and progression described in sufficient detail?

Yes

Is sufficient discussion included of the importance of the findings and their relevance to future understanding of disease processes, diagnosis or treatment?

Yes

Is the conclusion balanced and justified on the basis of the findings?

Yes

Reviewer Expertise:

Ocular surface and Cornea and anterior segment

We confirm that we have read this submission and believe that we have an appropriate level of expertise to confirm that it is of an acceptable scientific standard.
